# Imaging of primary vertebral tumors and tumor-like lesions in skeletally immature patients: a pictorial review

**DOI:** 10.1590/0100-3984.2025.0099

**Published:** 2026-09-27

**Authors:** Sergio Lopes Viana, Diógenes Diego de Carvalho Bispo, Bruno Beber Machado

**Affiliations:** 1 Tesla Diagnóstico por Imagem, Brasília, DF, Brazil; 2 Hospital da Criança de Brasília José Alencar, Brasília, DF, Brazil; 3 Dasa/Exame Medicina Diagnóstica, Brasília, DF, Brazil; 4 Instituto Dasa de Ensino e Pesquisa, São Paulo, SP, Brazil; 5 School of Medicine, University of Brasília, Brasília, DF, Brazil; 6 Gastren Clínica Médica e Radiológica, Iúna, ES, Brazil; 7 Clínica Radiológica Med Imagem, Cachoeiro de Itapemirim, ES, Brazil; 8 Clínica Radiológica Radial, Cachoeiro de Itapemirim, ES, Brazil; 9 Santa Casa de Misericórdia, Cachoeiro de Itapemirim, ES, Brazil; 10 Unimed Sul Capixaba, Cachoeiro de Itapemirim, ES, Brazil

**Keywords:** Spinal neoplasms, Bone neoplasms, Pediatrics, Magnetic Resonance Imaging, Tomography, X-ray computed, Osteomyelitis/physiopathology, Neoplasias da coluna vertebral, Neoplasias ósseas, Pediatria, Imageamento por ressonância magnética, Tomografia computadorizada por raios X, Osteomielite/fisiopatologia

## Abstract

Primary vertebral tumors and tumor-like lesions in skeletally immature patients are uncommon yet clinically consequential causes of pain, deformity, and neurological compromise. Compared with adults, pediatric lesions arise in a spine with open physes, evolving marrow composition, and substantial remodeling capacity—features that shape tumor biology and imaging appearance. This pictorial review summarizes malignant tumors, benign tumors, and tumor-like lesions of the pediatric spine, adopting a modality-first, compartment-aware approach. Useful highlights include vertebra plana in Langerhans cell histiocytosis, posterior-element bias in lesions such as osteoid osteoma/osteoblastoma, and the role of whole-body magnetic resonance imaging patterns in chronic nonbacterial osteomyelitis/chronic recurrent multifocal osteomyelitis. We also update terminology and concepts for entities such as vertebral hemangioma (venous malformation) and aneurysmal bone cyst. Throughout, we couple modality choice with pediatric anatomy and growth considerations, as well as illustrating the discussion with real-life cases from our teaching files.

## Abstract (pt)

Os tumores e pseudotumores vertebrais primários em pacientes esqueleticamente imaturos são incomuns, porém clinicamente relevantes como causas de dor, deformidade e comprometimento neurológico. Em relação aos adultos, as lesões pediátricas surgem em uma coluna com fises abertas, medula óssea em evolução e grande capacidade de remodelação—características que moldam a biologia tumoral e a aparência nas imagens. Esta revisão pictorial resume tumores malignos, tumores benignos e lesões pseudotumorais da coluna pediátrica, adotando uma abordagem centrada na modalidade e atenta ao compartimento. Destaques úteis incluem a vertebra plana na histiocitose de células de Langerhans, o predomínio de acometimento dos elementos posteriores em lesões como osteoma osteoide/osteoblastoma e o papel dos padrões de ressonância magnética de corpo inteiro na osteomielite não bacteriana crônica/osteomielite multifocal recorrente crônica, além da atualização de terminologia e conceitos em malformação venosa (“hemangioma” vertebral) e cisto ósseo aneurismático. Integramos as modalidades de imagem às particularidades anatômicas e de crescimento pediátricas e ilustramos a discussão com casos do nosso arquivo de ensino.

## INTRODUCTION

When children and adolescents present with spinal red flags—night pain, pain persisting beyond four weeks, focal neurological deficits, progressive spinal deformity, or systemic symptoms—imaging of the spine to screen for neoplastic and tumor-like etiologies is essential^**(^1^–^4^)**^. The skeletally immature spine differs from the adult spine in marrow composition, vascularity, and the presence of multiple open physes and synchondroses; these features influence tumor predilection (for example, a posterior-element bias), patterns of spread, signal characteristics, and, in some entities, a capacity for height reconstitution after collapse^**(^1^,^4^)**^. Initial localization (intradural vs. extradural; and vertebral body vs. neural arch) narrows the differential diagnosis and guides management^**(^4^)**^. In this pictorial review, we integrate modality choices with pediatric anatomy and growth considerations, illustrating the text with real-life cases drawn from our teaching files, rather than from a single institutional cohort. Table 1 summarizes the entities covered in this pictorial review, including typical age/peak incidence, vertebral predilection, key imaging findings, and the main differential diagnoses.

### What each modality adds

Conventional radiography remains the first look for deformity, collapse, and matrix calcifications and is the classic modality for identifying vertebra plana in Langerhans cell histiocytosis (LCH), though early findings may be subtle^**(^2^,^3^,^5^)**^. Computed tomography (CT) excels at matrix characterization—distinguishing “cloud-like” osteoid matrix from rings-and-arcs chondroid matrix—and at depicting cortical permeation and tiny calcifications; thin-section targeted reconstructions improve detection of a small nidus and support safe biopsy planning^**(^6^,^7^)**^. Magnetic resonance imaging (MRI) maps marrow infiltration, as well as epidural/paravertebral spread, and is indispensable for staging Ewing sarcoma and chordoma; edema-predominant benign lesions (osteoid osteoma/osteoblastoma) may obscure a nidus, inflammatory marrow can mimic tumor, and aggressive vascular lesions can be hypointense on T1-weighted imaging (T1WI)—the correlation with CT is often decisive^**(^3^,^5^,^8^–^15^)**^. Whole-body MRI (WBMRI) is preferred for baseline mapping and follow-up in chronic nonbacterial osteomyelitis/chronic recurrent multifocal osteomyelitis (CNO/CRMO) and LCH, capturing silent vertebral lesions and lesion-distribution patterns that facilitate the differential diagnosis and therapy monitoring^**(^16^–^19^)**^.

**Table 1:** Summary of primary vertebral tumors and tumor-like lesions in skeletally immature patients.

Entity	Typical age of occurrence	Vertebral predilection	MRI	Key features	Main differentials
Ewing sarcoma	10–20 years	Thoracic; lumbosacral (incl. the sacrum); body	T1 low; T2 high	Marrow replacement with permeative destruction; large paravertebral; epidural soft-tissue mass; may cause neurologic compromise	Osteomyelitis; primary vertebral lymphoma; LCH
Osteosarcoma	Adolescence	Thoracolumbar > sacrum (variable); body ± posterior elements	T1 low; T2 high	Aggressive bone destruction; osteoid; mineralized matrix (CT); soft-tissue extension; pathologic fracture possible	Ewing sarcoma; infection; metastasis
Chordoma	Rare in childhood & teenage	Craniovertebral junction; skull base–cervical more common in children; sacrococcygeal very rare; midline body	T1 low–intermediate; T2 very high	Midline lytic destructive mass; very high T2 with septations; avid enhancement; frequent epidural or paravertebral component	Chondrosarcoma; benign notochordal cell tumor
Primary vertebral lymphoma	Late childhood & adolescence	Thoracic and lumbar slightly more frequent; body	T1 low; T2 intermediate–high	Marrow replacement with relatively preserved height early; smooth, sheet-like epidural; paravertebral soft tissue; discs often preserved early	Ewing sarcoma; LCH; osteomyelitis
ABC	< 20 years	Posterior elements; neural arch; thoracic; lumbar (can be cervical)	T1 variable; T2 high	Expansile lytic lesion; multiple fluid–fluid levels; septal enhancement; may extend into vertebral body or spinal canal	Osteoblastoma; telangiectatic osteosarcoma; GCT
GCT of bone	Late adolescence & young adulthood (near/after skeletal maturity)	Sacrum >> mobile spine; often eccentric body	T1 low; T2 variable	Locally aggressive lytic lesion; heterogeneous signal and enhancement; may show ABC-like change; can invade adjacent vertebrae	Chordoma (sacrum); ABC
Osteoid osteoma	Teenage & young adulthood	Neural arch; posterior elements; lumbar > cervical; thoracic	T1 low; T2 variable	Small nidus (CT best) with marked reactive sclerosis; disproportionate marrow; soft-tissue edema on MRI; nocturnal pain responsive to NSAIDs	Osteoblastoma
Osteoblastoma	Late childhood, teenage & young adulthood	Neural arch; posterior elements; cervical and lumbar common	T1 low; T2 variable	Larger than osteoid osteoma; expansile lytic lesion with variable mineralization; prominent enhancement and edema; may cause scoliosis or neurologic symptoms	Osteoid osteoma; aneurysmal bone cyst; osteosarcoma (aggressive variant)
Osteochondroma	Childhood & adolescence	Posterior elements (pedicle; lamina); cervical and upper thoracic typical	T1 low; T2 high (cartilage cap)	Exostosis with continuity of cortex and medullary canal; cartilage cap assessment on MRI; can be multiple in HME	Other posterior-element tumors (e.g., osteoblastoma); fibro-osseous lesions
Vertebral hemangioma (venous malformation)	Uncommon in childhood	Thoracic > lumbar; body	T1 high; T2 high	Typical: T1 high; thickened trabeculae on T2WI (corduroy or polka-dot on CT). Aggressive: T1 low, expansion, epidural extension and neurologic compromise	Metastasis; lymphoma
LCH	< 10 years	Thoracic predominance; body	T1 low; T2 high	Vertebra plana with preserved discs; paraspinal soft-tissue component may be present; multifocal skeletal disease possible	Osteomyelitis; Ewing sarcoma; metastasis
CNO; CRMO	7–15 years	Thoracic; lumbar; multifocal; body (endplates; corners) ± posterior elements	T1 low; T2/STIR high	Multifocal inflammatory lesions; no abscess; necrosis; recurrent course; may mimic infection; malignancy; WBMRI helps map disease	Infection; LCH; Ewing sarcoma
Fibrous dysplasia	Childhood & adolescence	Variable; spinal involvement uncommon	T1 low; T2 variable	Ground-glass matrix (CT); bony expansion and cortical thinning; MRI findings variable depending on cellularity & fibrous content	Simple bone cyst; other tumors

T1 low, hypointense signal on T1WI; T2 high, hyperintense signal on T2WI; T1 low–intermediate, low–intermediate signal intensity on T1WI; T2 very high, extremely high signal intensity on T2WI; T2 intermediate–high, intermediate–high signal intensity on T2WI; T1 variable, variable signal intensity on T1WI; T2 variable, variable signal intensity on T2WI; NSAIDs, nonsteroidal anti-inflammatory drugs; HME, hereditary multiple exostoses; STIR high, hyperintense signal on STIR sequences.

Nuclear medicine is sensitive but nonspecific for multifocal disease; in children, WBMRI is favored when feasible^**(^16^–^18^)**^. Biopsy planning should leverage cross-sectional imaging to minimize the number of passes and avoid contaminating future resection planes, coordinated with the surgical team^**(^3^,^6^,^12^,^13^,^20^)**^.

### Clinical and imaging presentation

#### Malignant tumors

##### Ewing sarcoma

At-a-glance—Demographic profile: children and adolescents; Segment: sacrum and thoracolumbar most common; Portion: vertebral body, often with a large soft-tissue mass.

Ewing sarcoma is the most common primary malignant vertebral tumor in children and adolescents, found mainly in the sacrum or thoracolumbar spine^**(^12^–^14^,^20^)**^. Imaging typically shows permeative lysis with cortical destruction (Figure 1), often with a disproportionately large soft-tissue mass on CT or MRI^**(^3^,^5^,^9^,^21^)**^. In the sacrum, the lesions may cross the sacroiliac joint space, with contiguous involvement of the adjacent ilium^**(^21^)**^, as shown in Figure 2. On MRI, marrow replacement shows a low-intensity signal on T1WI, shows a high-intensity signal on fluid-sensitive sequences, and is heterogeneously enhancing; epidural extension and canal compromise are common^**(^11^,^12^)**^. Osteomyelitis and lymphoma can mimic Ewing sarcoma; the combination of aggressive osseous destruction and bulky soft-tissue disease favors Ewing sarcoma^**(^3^,^5^,^11^,^12^)**^. When feasible, wide or en bloc margins improve local control, although it is challenging to achieve safe margins in the spine and pelvis; pediatric planning must weigh stabilization against growth^**(^20^)**^.

**Figure 1:**
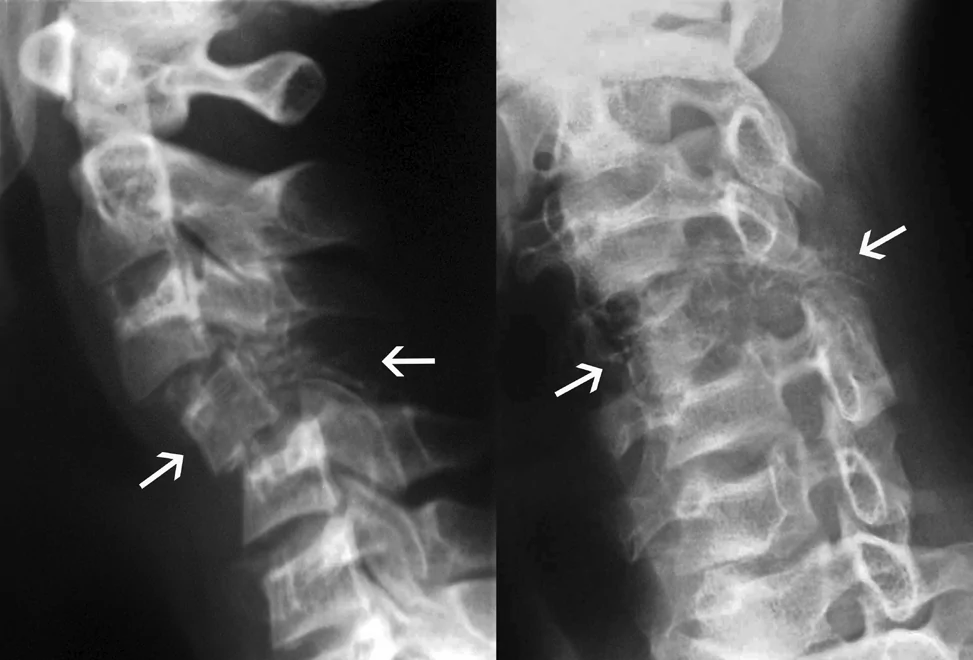
Cervical spine radiographs of a 12-year-old boy with Ewing sarcoma. Note the permeative osteolytic lesion involving the C4 vertebral body and posterior elements (straight arrows), with a radiolucent matrix and subluxation at C3–C4 and C4–C5.

**Figure 2:**
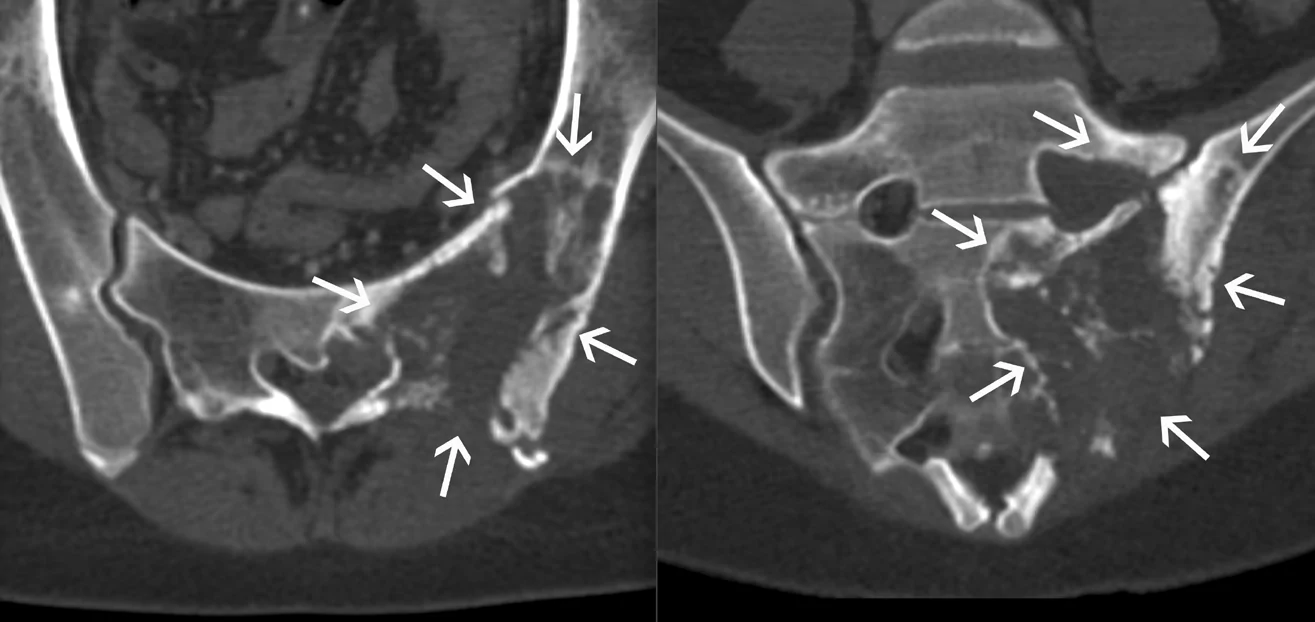
Axial and coronal CT images of a 16-year-old girl with Ewing sarcoma of the sacrum displaying a large aggressive lesion (straight arrows) with a wide zone of transition centered in the left sacral ala, causing marked osteolysis, crossing the sacroiliac joint, and resulting in osseous destruction of the adjacent ilium.

##### Osteosarcoma

At-a-glance—Demographic profile: adolescents; Segment: thoracolumbar > sacrum (variable); Portion: vertebral body, with or without posterior elements.

Although spinal osteosarcoma is uncommon in children, it is aggressive when it occurs^**(^12^,^20^,^22^)**^. Computed tomography shows a mixed lytic–sclerotic lesion with a “cloud-like” osteoid matrix or, less often (in osteoblastic forms), an ivory vertebra^**(^9^,^12^)**^. The use of MRI allows the delineation of intramedullary extent, cortical breakthrough, epidural spread, and soft-tissue masses; the mineralized component of the tumor—whether osseous or in the soft tissues—appears hypointense on T1WI and T2WI^**(^12^)**^, as illustrated in Figures 3 and 4. Telangiectatic osteosarcoma may show fluid–fluid levels and can mimic an aneurysmal bone cyst; solid enhancing components, an aggressive periosteal reaction, and permeative margins support osteosarcoma^**(^5^,^9^,^12^–^14^,^22^)**^. Surgical planning must balance decompression/stabilization with oncologic control; local recurrence is lower with wide margins when achievable^**(^12^,^20^)**^.

**Figure 3:**
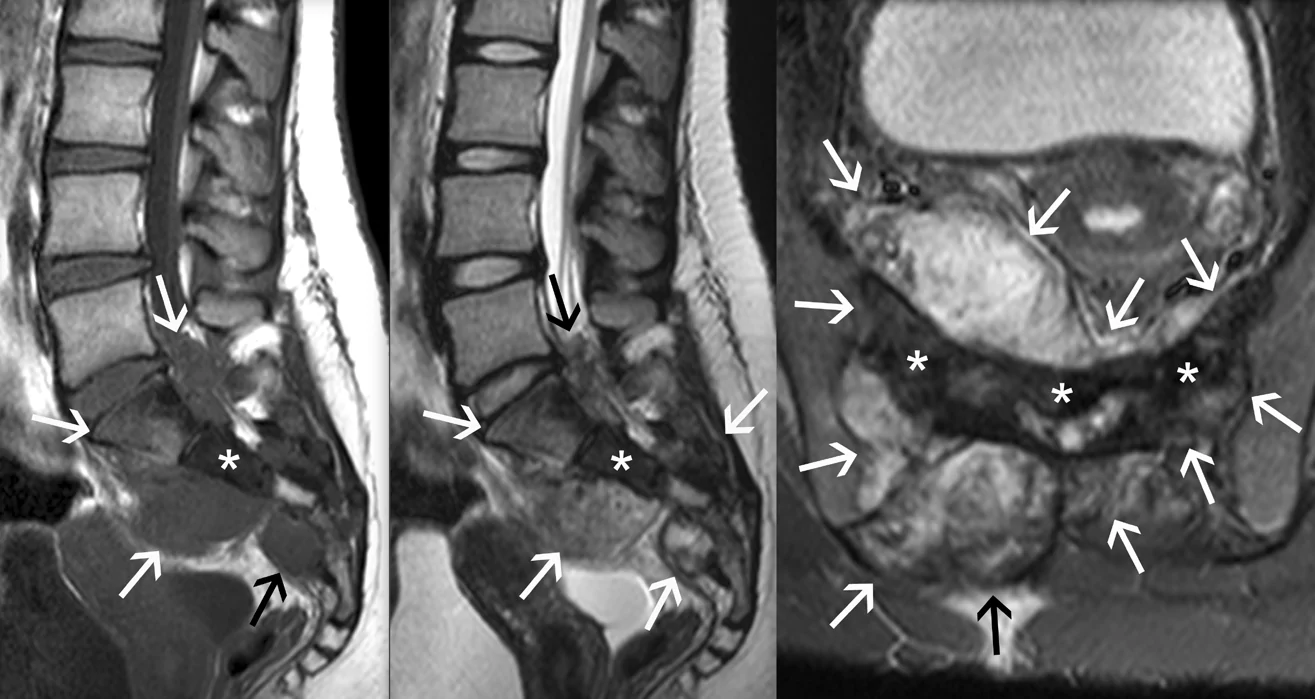
MRI of a 15-year-old girl with sacral osteosarcoma. Sagittal T1WI (left), sagittal T2WI (center), and axial contrast-enhanced fat-suppressed T1WI (right), showing a large expansile mass centered at S2 (straight arrows) with diffuse osseous infiltration, destruction of the posterior portion of the right ilium, and an extensive soft-tissue component extending into the presacral space and spinal canal. Contrast enhancement is more conspicuous in the soft-tissue component. Areas of low signal intensity across all sequences (asterisks) represent mineralized osteoid matrix.

**Figure 4:**
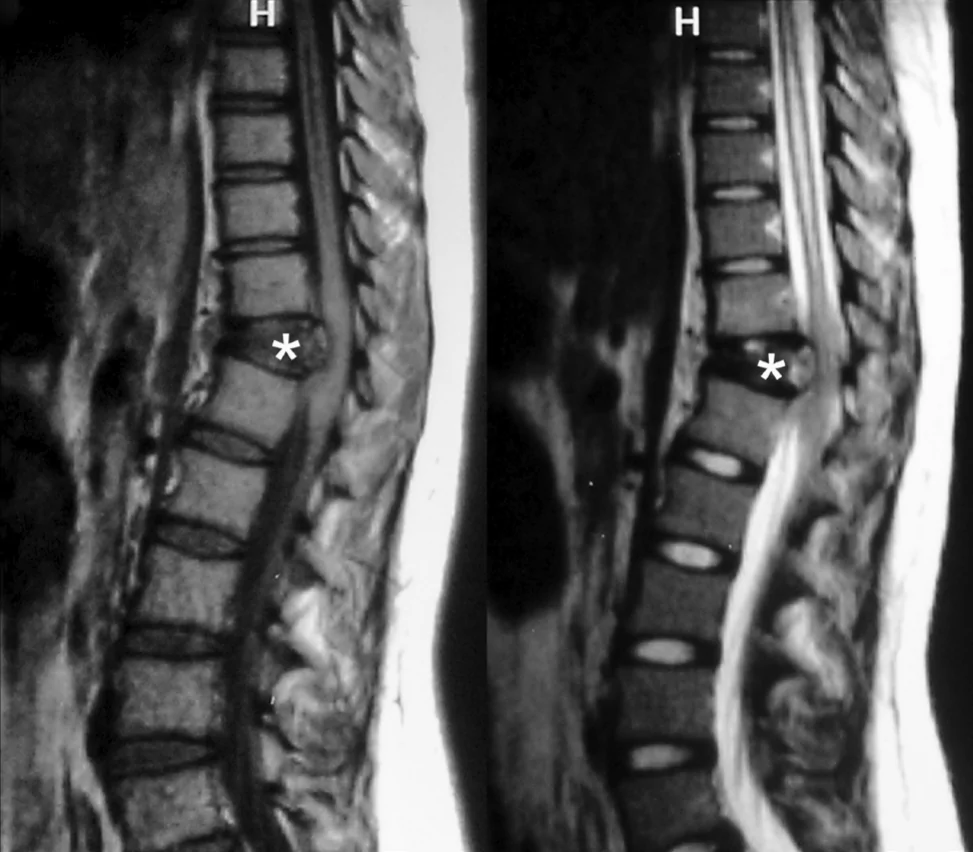
MRI of the thoracolumbar spine in a 15-year-old girl with vertebral osteosarcoma. Sagittal T1WI (left) and T2WI (right) demonstrating marked collapse of the T12 vertebral body, which shows heterogeneous signal intensity in the marrow; areas of hypointensity on both sequences reflect mineralized tumor matrix (asterisks). Note the posterior wall bulging compressing the ventral spinal cord, with dilation of the central canal above that level.

##### Chordoma

At-a-glance—Demographic profile: rare in children/adolescents, with an overall bimodal age distribution (small early-age cluster, larger adult peak); Segment: sacrum > clivus > mobile spine and, in children, sacrococcygeal disease is very rare compared with craniovertebral (skull base/cervical) involvement; Portion: midline of the vertebral body (central).

Chordoma arises from notochordal remnants. In the general population, it more often affects the sacrum than the craniovertebral junction. However, in children, sacrococcygeal chordoma is distinctly uncommon; instead, skull-base and cervical disease account for the clear majority^**(^23^)**^. A CT scan shows central lysis with faint calcifications^**(^12^)**^, as depicted in Figure 5. An MRI scan depicts a lobulated mass that shows very high-intensity signal on T2WI, with thin hypointense septa and heterogeneous enhancement, commonly with epidural/paravertebral extension^**(^11^,^12^,^24^)**^. The main differential diagnoses are as follows^**(^11^,^12^,^24^)**^: benign notochordal cell tumor, characterized by no cortical breach or soft-tissue mass; giant cell tumor (GCT), characterized by a less gelatinous appearance on T2WI, often with aneurysmal bone cyst (ABC)-like change; and chondrosarcoma, characterized by a rings-and-arcs pattern of calcification.

**Figure 5:**
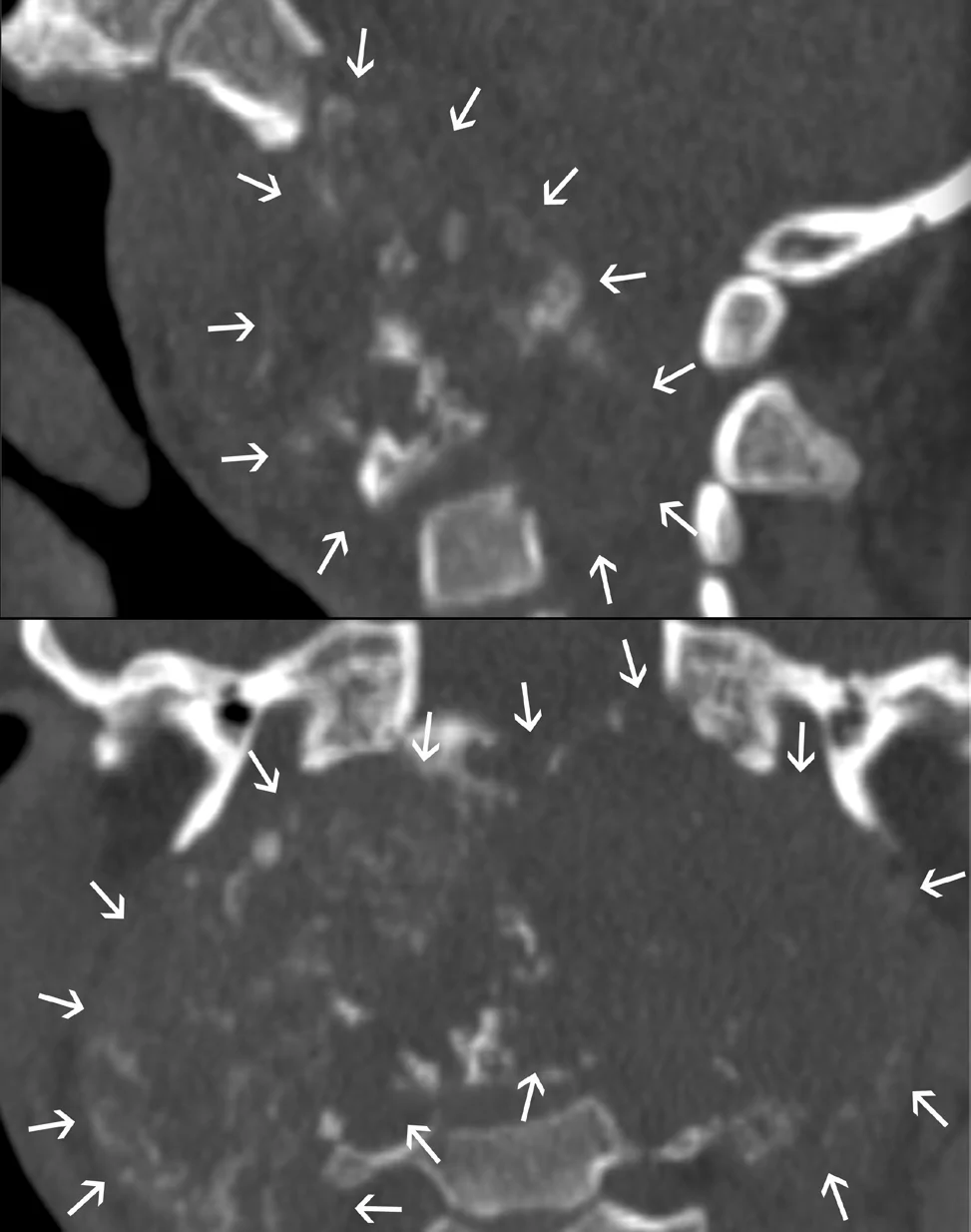
Craniovertebral junction chordoma in an 11-year-old boy. Sagittal and coronal reformatted CT images (left and right, respectively) showing a bulky expansile lesion (straight arrows) with a mineralized matrix causing destruction of the inferior clivus, the anterior arch of C1, and most of the C2 body. A large soft-tissue component extends laterally, into the vertebral canal, to the skull base (anterior to the brainstem), and prevertebrally, with bulging of the posterior pharyngeal wall.

##### Primary vertebral lymphoma

At-a-glance—Demographic profile: children/adolescents; Segment: thoracic and lumbar slightly more common; Portion: vertebral body with smooth, sheet-like epidural/paravertebral soft tissue, often with early preservation of vertebral height and adjacent discs.

In children, primary vertebral lymphoma (primary bone lymphoma of the spine) typically presents with insidious spinal pain, sometimes with radicular symptoms or early neurologic compromise. On MRI, marrow replacement shows diffuse, homogeneous hypointensity on T1WI and hyperintensity on short-tau inversion recovery (STIR) sequences, with relatively uniform enhancement and a smooth, plaque-like epidural or paravertebral soft-tissue component that may be disproportionately large relative to the degree of cortical destruction (Figure 6); vertebral height and adjacent discs are often preserved in the early stages^**(^3^,^4^,^11^,^12^,^25^)**^. Restricted diffusion is common in the marrow and soft tissue, and CT may show subtle lysis or sclerosis with minimal periosteal reaction^**(^11^,^12^,^25^)**^. Compared with Ewing sarcoma, cortical permeation and aggressive periosteal change are typically less pronounced, and the soft-tissue envelope is more uniform^**(^3^,^4^,^11^,^12^)**^. Although fluorodeoxyglucose positron emission tomography/computed tomography helps confirm osseous unifocality, exclude nodal/visceral disease, and establish a baseline for response assessment, histopathologic confirmation remains mandatory^**(^11^,^12^,^25^)**^.

**Figure 6:**
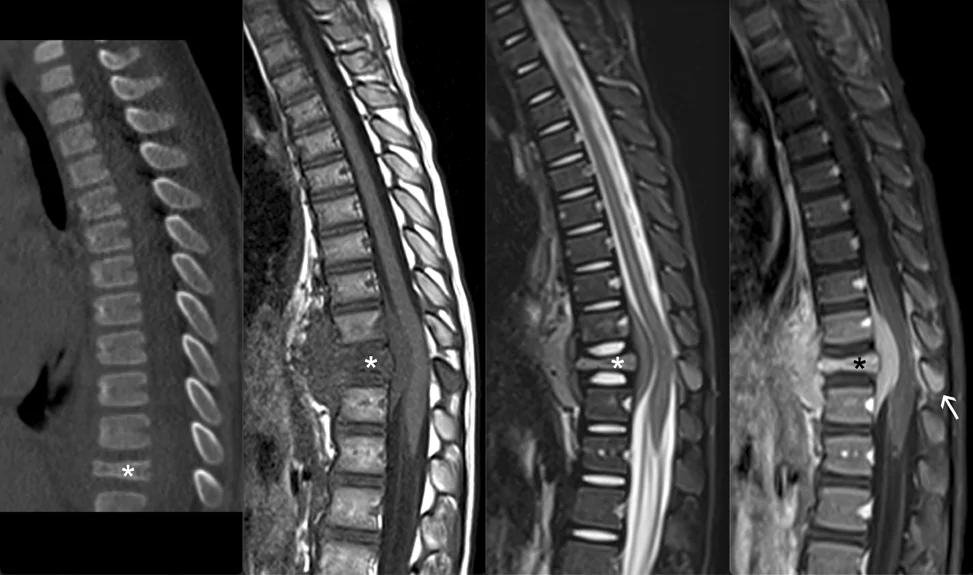
B-lymphoblastic lymphoma of T11 in a 4-year-old boy. Sagittal reformatted CT image (first, starting from the left), sagittal T1WI (second), STIR sequence (third), and contrast-enhanced fat-suppressed T1WI (fourth), showing partial collapse of the T11 vertebral body, which is mildly sclerotic on CT, hypointense on T1WI, hyperintense on the STIR sequence, and avidly enhancing, with preservation of the adjacent intervertebral discs (asterisks). Note the prevertebral and epidural soft-tissue masses, which show intense enhancement, compressing the cord with signal changes indicative of compressive myelopathy. The T11 spinous process is also infiltrated and shows enhancement (straight arrow).

#### Benign tumors

##### ABC

At-a-glance—Demographic profile: first two decades of life; Segment: thoracic/lumbar (can be cervical); Portion: neural arch/posterior elements.

An ABC is now regarded as a true neoplasm driven by the ubiquitin-specific protease 6 (USP6) gene rearrangements; USP6 encodes a deubiquitinating enzyme whose overexpression promotes cyst formation and local bone remodeling^**(^26^,^27^)**^. The previously termed “secondary ABC,” denoting cystic-hemorrhagic change within another tumor, is best called “ABC-like changes” in contemporary World Health Organization usage; USP6 testing—by fluorescence in situ hybridization or next-generation sequencing—supports primary (USP6-positive) ABC and is typically negative in ABC-like change^**(^26^–^28^)**^. Conventional radiographs and CT usually show an expansile, septated lytic lesion with cortical thinning/remodeling and internal trabeculations; CT may also depict fluid–fluid levels and small dependent layering, which makes it valuable for preprocedural planning^**(^2^,^12^,^26^,^27^)**^. An MRI scan demonstrates fluid–fluid levels, internal septa, and peripheral/septal enhancement, with scant solid tissue in typical cases (Figure 7); when substantial solid enhancing components are present, consider ABC-like changes over another tumor or the rare solid variant of ABC, which remains uncommon but has been reported in children and adolescents—including in the spine^**(^12^,^27^,^28^)**^, as shown in Figure 8.

**Figure 7:**
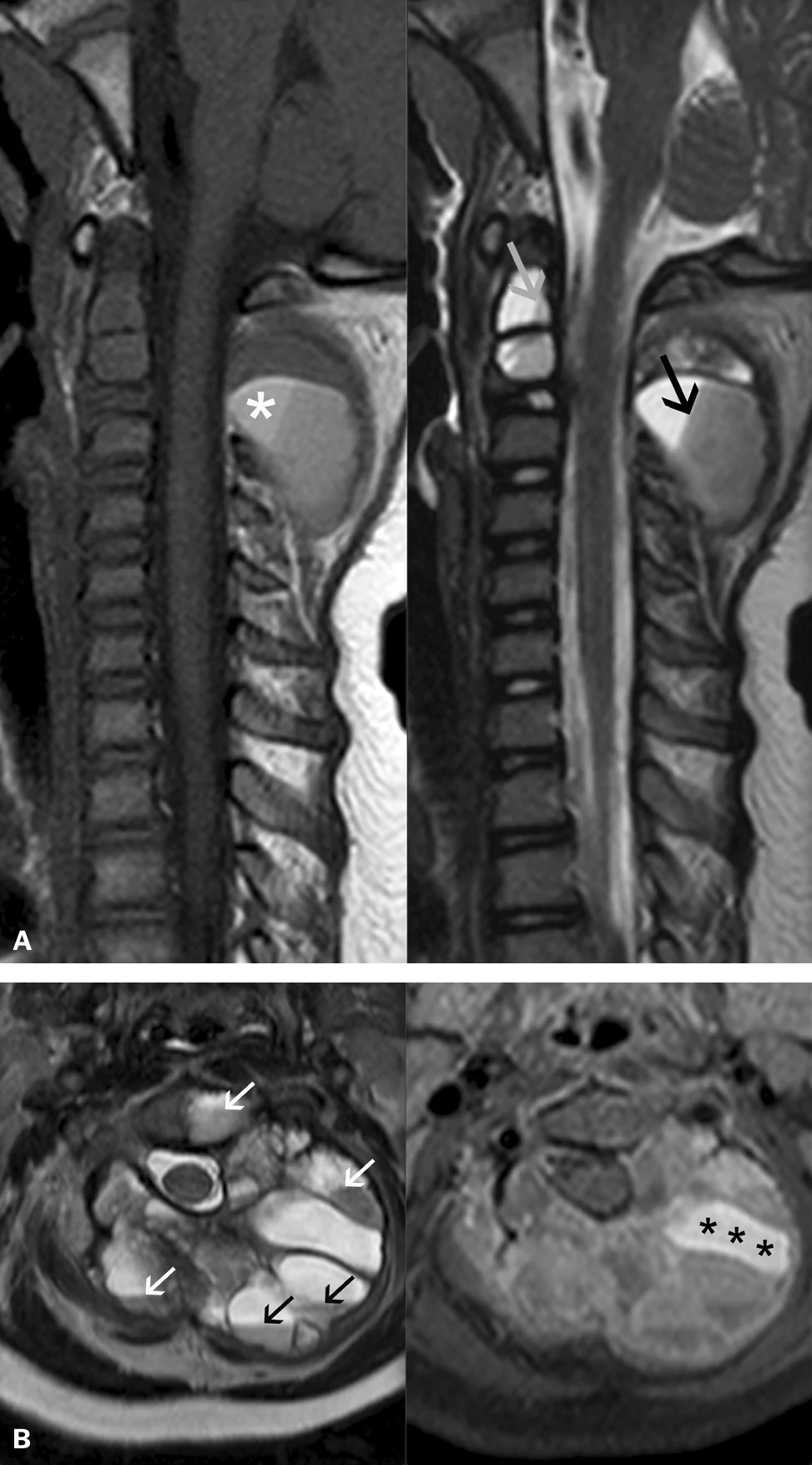
ABC of C2 in a 5-year-old girl. Sagittal T1WI and T2WI (**A**), together with axial T2WI and contrast-enhanced fat-suppressed T1WI (**B**), showing a large expansile lesion arising from the posterior elements with substantial involvement of the vertebral body. Multiple septated cystic spaces show fluid–fluid levels (straight arrows) and areas of intrinsic hyperintensity on T1WI, consistent with hemorrhagic content (asterisks). Note the peripheral and septal enhancement.

**Figure 8:**
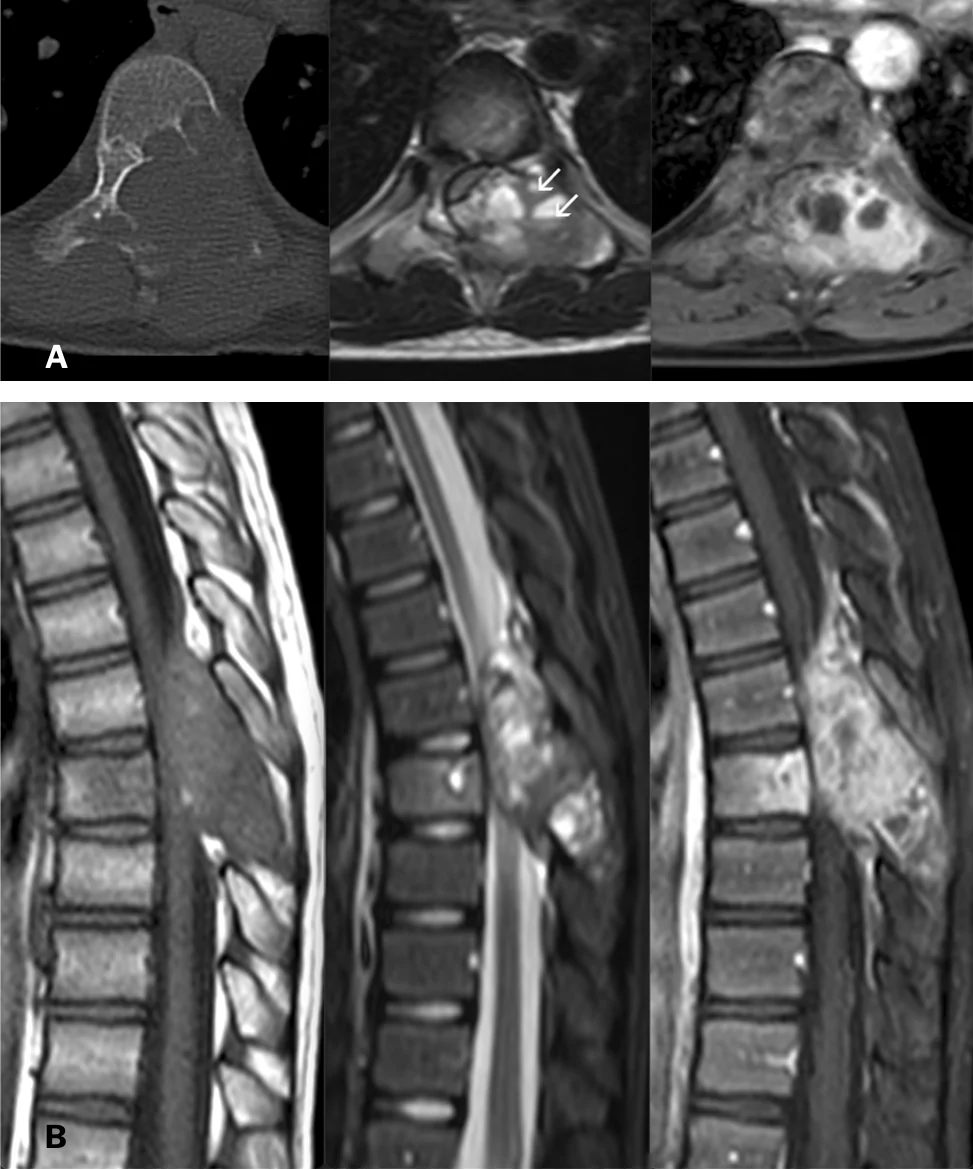
Solid variant of ABC of T8 in an 11-year-old girl. Axial CT, axial T2WI MRI sequence, and contrast-enhanced fat-suppressed T1WI MRI sequence (**A**), together with sagittal T1WI, STIR, and contrast-enhanced fat-suppressed T1WI MRI sequences (**B**), showing a lytic lesion centered in the left posterior elements with extension into the vertebral body that invaded the spinal canal and markedly displaced the spinal cord. In addition to the characteristic cystic component with fluid–fluid levels (straight arrows) and peripheral/septal enhancement, there is a prominent avidly enhancing solid component. Pathology and immunohistochemistry confirmed the diagnosis, and the lesion was USP6-positive.

##### GCT of bone

At-a-glance—Demographic profile: skeletally mature/near-mature individuals (late teens/young adults); Segment: sacrum far more common than the mobile spine; Portion: vertebral body (often eccentric) with possible posterior extension.

A GCT is locally aggressive^**(^11^,^12^)**^. In the mobile spine, aggressive cases are typically osteolytic with cortical destruction, are vertebral body-centered, and, in many cases, show epidural extension with pathologic fracture/collapse; cystic areas are common, and fluid–fluid levels occur in a subset of cases^**(^29^)**^, as illustrated in Figure 9. Invasion of an adjacent vertebra has been reported and should be actively sought on sagittal and axial imaging^**(^30^)**^. In the sacrum, GCTs are often eccentric and may extend across the sacroiliac joint into the ilium, a useful discriminator from midline sacral chordoma^**(^31^)**^. Although mobile-spine and sacral lesions may both display ABC-like change and avid enhancement, a midline sacral chordoma—with very high signal intensity on T2WI and a midline epicenter—remains the key differential^**(^11^,^12^,^24^,^31^)**^.

**Figure 9:**
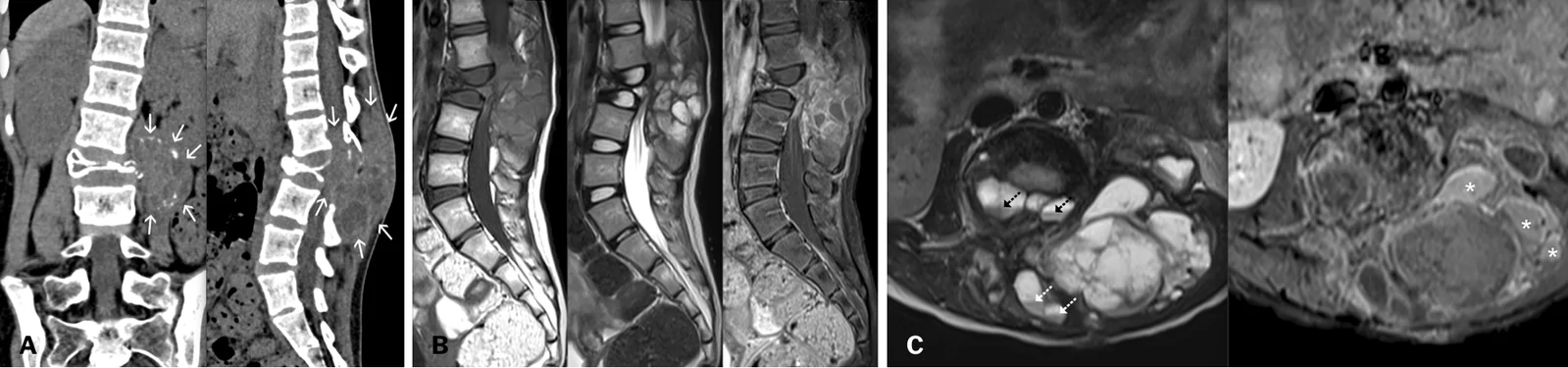
GCT in a 12-year-old girl. In **A**, coronal and sagittal CT images showing marked asymmetric collapse of L3 and a large mass arising from the posterior elements with a bulky left-sided soft-tissue component (straight arrows) extending into the spinal canal; there was no tumor matrix calcification (only peripheral shell-like osseous fragments), although there were internal areas of hypodensity. Sagittal T1WI, T2WI, and contrast-enhanced fat-suppressed T1WI (**B**), together with axial T2WI and contrast-enhanced fat-suppressed T1-WI (**C**), showing near-complete canal obliteration by a heterogeneously enhancing solid component with additional cystic areas, some with fluid–fluid levels (dashed arrows), with or without hemorrhagic content that is intrinsically hyperintense on T1WI (asterisks), showing peripheral/septal enhancement, corresponding to ABC-like changes.

##### Osteoid osteoma

At-a-glance—Demographic profile: teens, predominantly males; Segment: lumbar > cervical/thoracic; Portion: neural arch/posterior elements.

Osteoid osteoma classically presents with nocturnal pain relieved by nonsteroidal anti-inflammatory drugs and is a leading cause of painful scoliosis when spinal, often with convexity away from the lesion and associated facet synovitis^**(^2^)**^. A CT scan is decisive for identifying the < 1.5–2.0 cm nidus—frequently with central mineralization—within reactive cortical/sclerotic bone (Figure 10); thin, targeted reconstructions maximize conspicuity and guide ablation^**(^5^–^7^)**^. The use of MRI can be pitfall-prone in the spine: extensive marrow and soft-tissue edema, joint effusion, and enhancement can overwhelm or obscure a tiny nidus; recognition of a focal enhancing dot within edema is helpful, but correlative CT is often mandatory^**(^10^,^14^,^32^)**^. Although bone scintigraphy typically shows focal hyperemia and uptake (often a “double-density” sign), WBMRI is increasingly favored in children when the differential diagnosis includes multifocal processes to limit ionizing radiation^**(^16^–^19^,^32^)**^.

**Figure 10:**
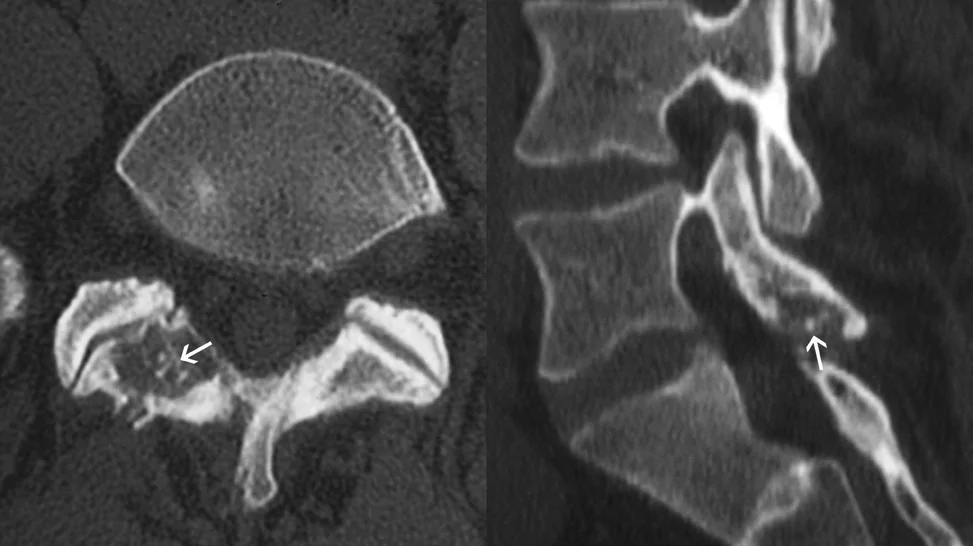
Axial and sagittal CT images of the lumbar spine in a 16-year-old boy, showing classic features of osteoid osteoma of the right inferior articular facet of L5: a small, well-circumscribed radiolucent lesion with a centrally mineralized nidus (straight arrows), together with osseous expansion and adjacent cortical irregularity.

##### Osteoblastoma

At-a-glance—Demographic profile: older children/teens, young adults; Segment: cervical and lumbar predilection; Portion: neural arch/posterior elements.

Osteoblastoma spans a broad spectrum of presentations on imaging, from lesions that appear giant osteoid osteoma-like (well-circumscribed, dominant nidus-like core with surrounding sclerosis, as in Figure 11) to frankly aggressive tumors with cortical breakthrough, epidural/paravertebral extension, and prominent soft-tissue reaction^**(^11^,^12^)**^, as depicted in Figure 12. Approximately 30–40% arise in the spine, usually from the neural arch with potential vertebral body extension, and canal/foraminal compromise often drives presentation^**(^5^,^11^,^12^)**^. An osteoblastoma is heterogeneous on fluid-sensitive MRI sequences with avid, variable enhancement; marrow/soft-tissue edema can mask the core lesion, so CT correlation is essential to demonstrate internal osteoid matrix and any coarse calcifications^**(^11^,^12^,^14^,^33^)**^, as illustrated in Figure 11. There can be ABC-like change, which may complicate differentiation from primary ABC and telangiectatic osteosarcoma; attention to solid enhancing tumor, matrix, and periosteal response helps avoid misclassification^**(^11^,^12^,^33^)**^.

**Figure 11:**
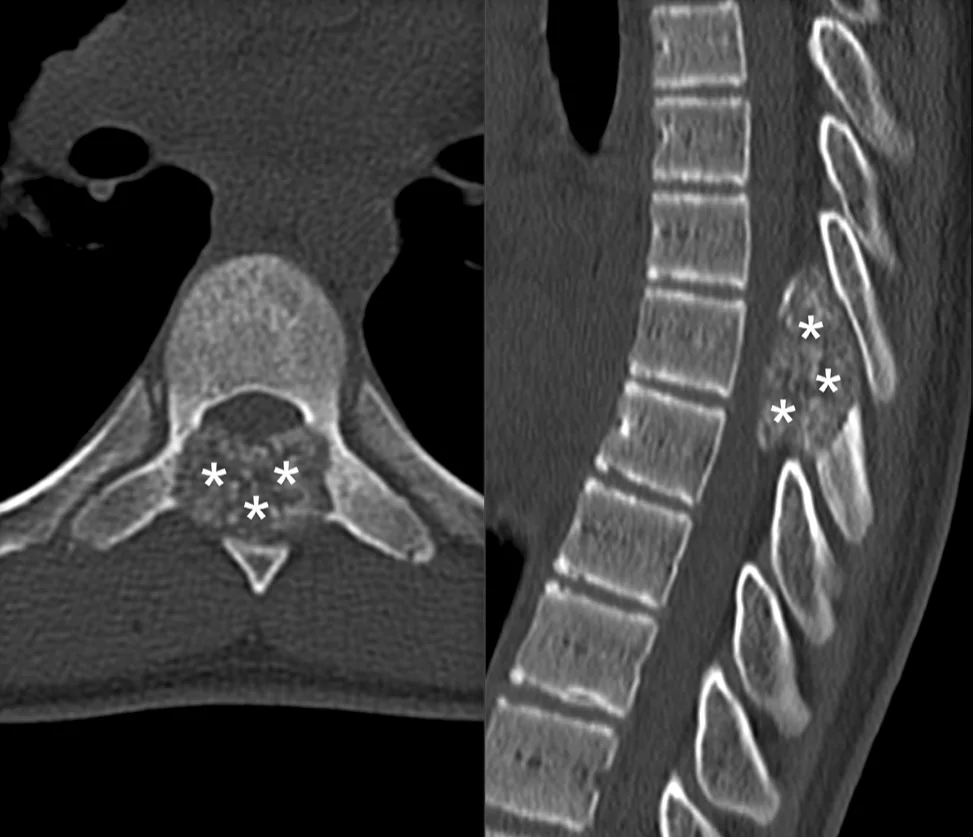
Osteoblastoma of T7 in an 11-year-old girl. Axial and sagittal CT images showing a coarsely mineralized expansile lesion (asterisks) broadly involving the posterior elements, with sharply defined borders, extending into the spinal canal and causing marked canal narrowing with posterior cord compression.

**Figure 12:**
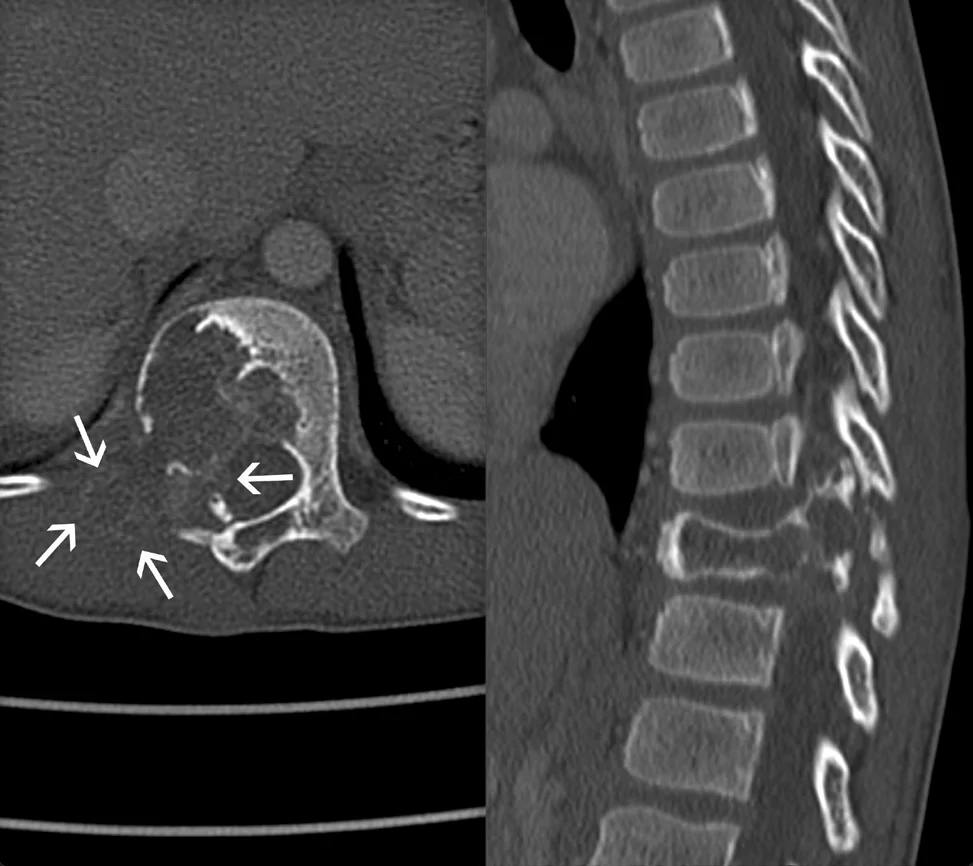
Aggressive osteoblastoma of T11 in an 8-year-old boy. Axial and sagittal CT images showing a lytic lesion involving the right posterior elements and most of the vertebral body (with moderate collapse), cortical breakthrough, and a soft-tissue component (straight arrows) extending into the paravertebral muscles and spinal canal, without appreciable matrix mineralization.

##### Osteochondroma

At-a-glance—Demographic profile: children/adolescents, with a higher risk in hereditary multiple exostoses; Segment: cervical and upper thoracic typical; Portion: neural arch/posterior elements (pedicle, lamina, spinous process).

Spinal osteochondromas show cortical and medullary continuity with the parent bone on CT and a T2-bright cartilage cap on MRI (Figure 13); lesions arise from the pedicle, lamina, or spinous process and can encroach upon the canal or foramina, producing radiculopathy or myelopathy^**(^2^,^10^)**^. Bursitis and reactive edema around the cap can mimic aggression, although a lack of destructive change and confirmation of continuity favor a benign diagnosis^**(^2^,^10^)**^. In children and adolescents, cartilage caps can be physiologically thicker during active growth; a threshold of > 3 cm (≈ 30 mm) is more commonly used as a threshold for malignant transformation, given that values up to ≈ 2–3 cm can be observed in skeletally immature patients^**(^34^,^35^)**^. In contrast, in skeletally mature patients, a threshold of > 2 cm is typically used to raise suspicion.

**Figure 13:**
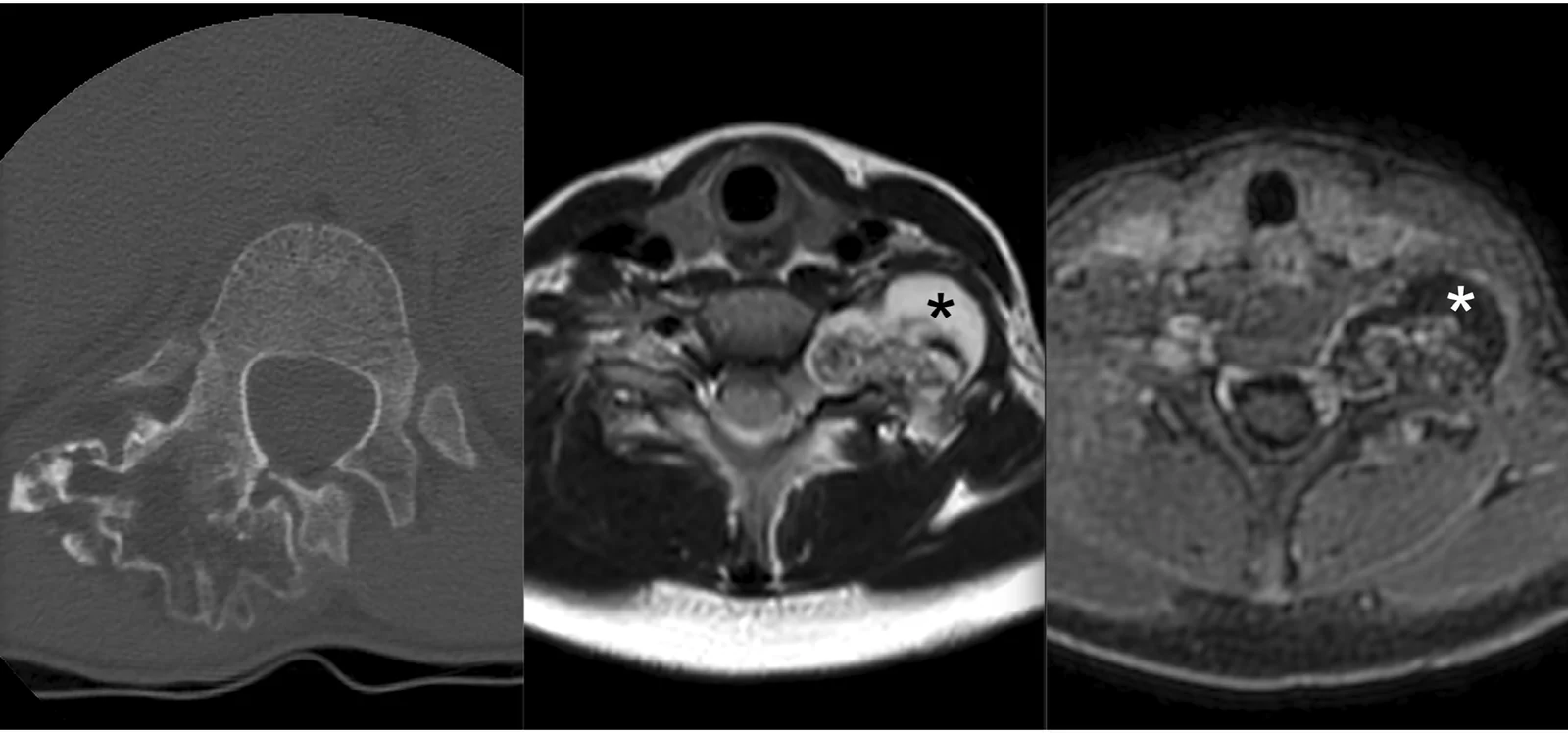
Vertebral osteochondromas in two separate patients. Although corticomedullary continuity with the host bone is usually best appreciated on CT (left image: large osteochondroma of the T12 posterior elements in a 12-year-old girl), MRI uniquely demonstrates and permits assessment of the cartilage cap (asterisks in the center image and the image on the right). Axial T2WI (center) and contrast-enhanced fat-suppressed T1WI (right), showing osteochondroma arising from the left C7 transverse process in an 11-year-old boy.

##### Vertebral hemangioma

At-a-glance—Demographic profile: uncommon in children; Segment: thoracic > lumbar; Portion: vertebral body.

Vertebral hemangiomas—biologically venous malformations per the International Society for the Study of Vascular Anomalies—remain widely labeled “hemangiomas” in daily practice^**(^36^)**^. They consist of vascular channels interlaced with thickened vertical trabeculae, producing the “polka-dot” sign on axial CT and vertical striations on sagittal/coronal images^**(^5^,^10^)**^, as illustrated in Figure 14. Aggressive variants can be hypointense on T1WI, hyperintense on T2WI, and avidly enhancing, with cortical erosion and epidural/paravertebral extension (Figure 15a); preservation of vertical trabeculae on CT (Figure 15b) favors a venous malformation over malignancy^**(^13^,^14^,^37^,^38^)**^.

**Figure 14:**
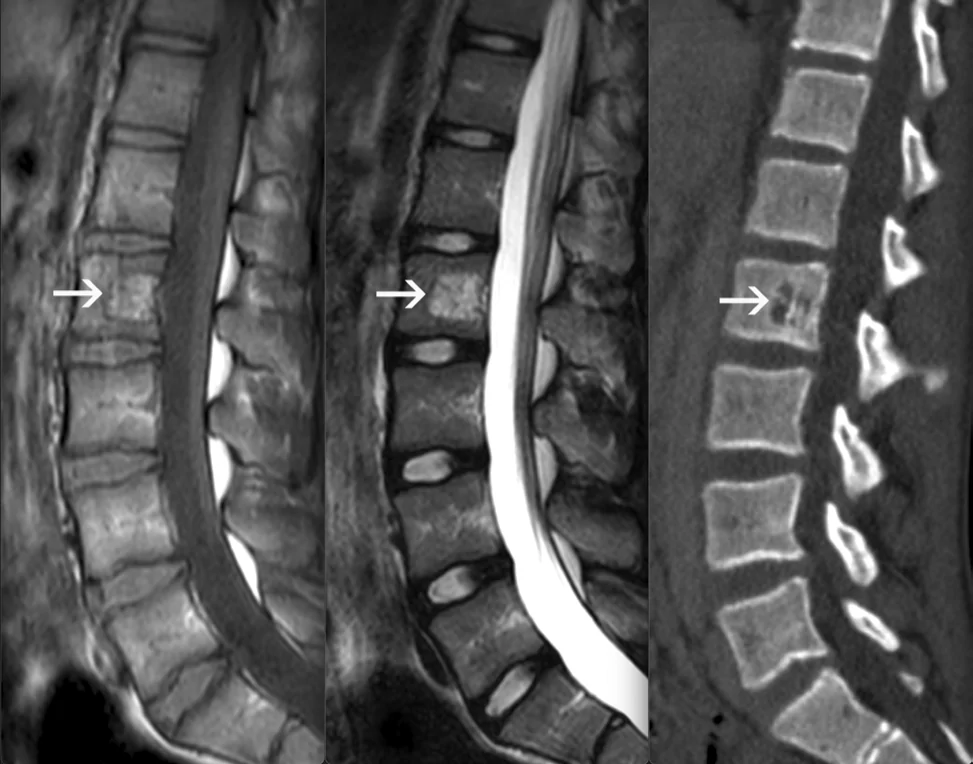
Typical L2 vertebral hemangioma in a 16-year-old boy. Straight arrows in each image indicate a well-defined nodular lesion, characterized by fat-like high signal intensity on T1WI (left) and T2WI (center), and by thickened vertical trabeculae with alternating hypodense stroma—the corduroy sign—on sagittal CT (right).

**Figure 15:**
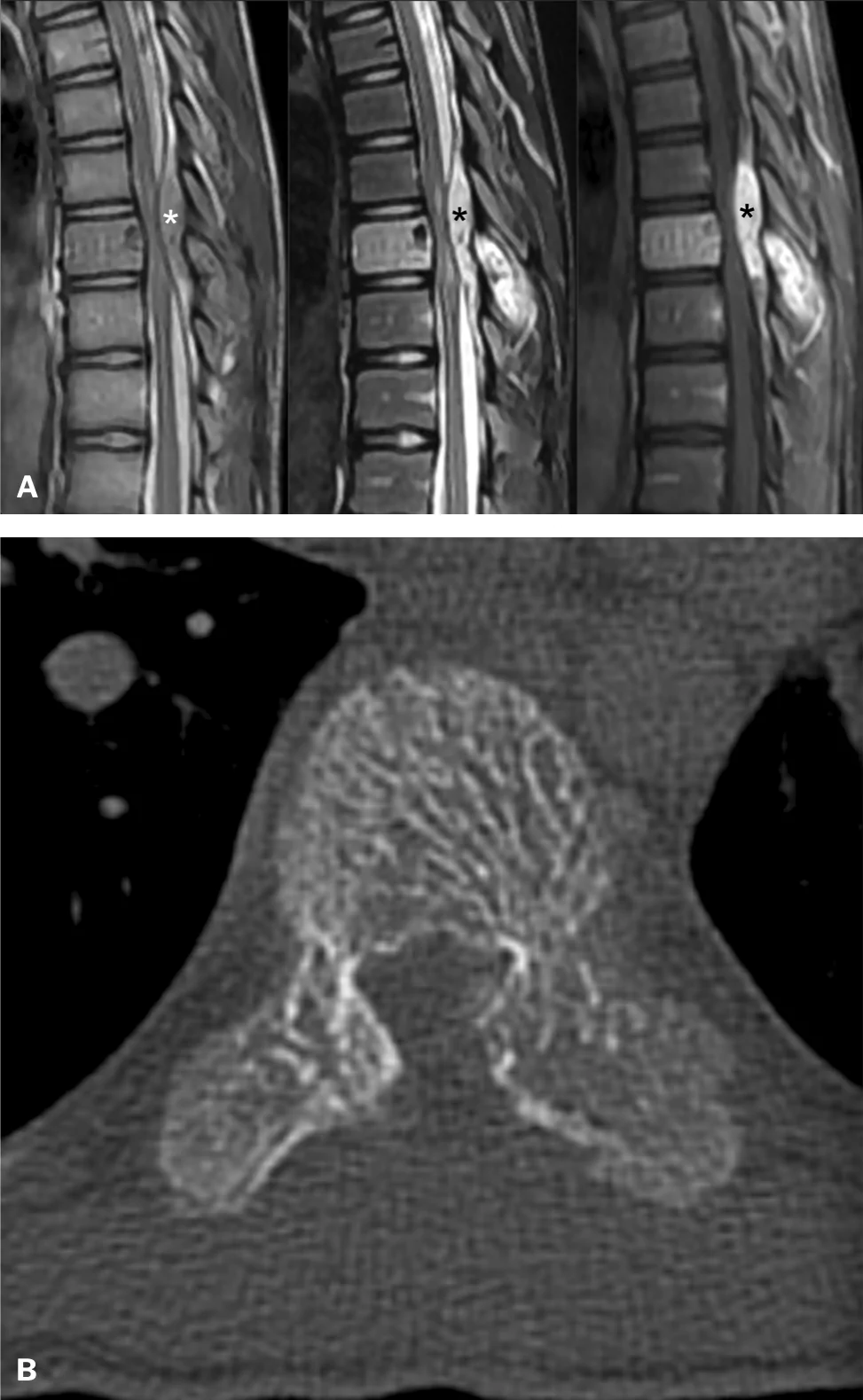
Aggressive variant of vertebral hemangioma in a 15-year-old boy. In **A**, sagittal T1WI, STIR, and contrast-enhanced fat-suppressed T1WI sequences showing mild osseous expansion and diffuse marrow signal abnormality in the T9 vertebral body and spinous process (intermediate on T1WI, hyperintense on a STIR sequence), with avid enhancement. Note the posterior epidural mass of similar signal intensity and enhancement (asterisks), causing posterior cord compression with features indicative of compressive myelopathy. Longitudinal striations with low signal intensity can be seen traversing the abnormal marrow. In **B**, a postoperative CT scan better depicts preserved, thickened trabeculae interspersed with hypodense stroma throughout the vertebral body and enlarged transverse processes. Note the changes indicative of decompressive laminectomy.

#### Tumor-like lesions

##### LCH

At-a-glance—Demographic profile: young children (peak at early school age); Segment: thoracic; Portion: vertebral body (vertebra plana), discs preserved.

The leading cause of vertebra plana in children is LCH, typically with preserved intervertebral discs and a relatively small paravertebral/epidural component compared with that seen in malignant collapse^**(^3^,^41^)**^. Conventional radiographs may show an evolving anterior wedge that progresses to complete collapse (vertebra plana), as shown in Figure 16, whereas CT confirms lysis, defining cortical integrity and any small epidural component when MRI is not immediately available^**(^5^,^11^,^13^,^41^)**^, as illustrated in Figure 17. An MRI scan shows a hypointense signal on T1WI and a hyperintense fluid-sensitive signal within the vertebral body, with variable enhancement and with the posterior elements often spared (Figure 18); over time, partial height reconstitution is common and supports conservative management when neurologically safe^**(^5^,^11^,^13^,^41^)**^. Because LCH can be multifocal, WBMRI helps detect additional skeletal sites and tailor follow-up in a radiation-free manner^**(^16^)**^.

**Figure 16:**
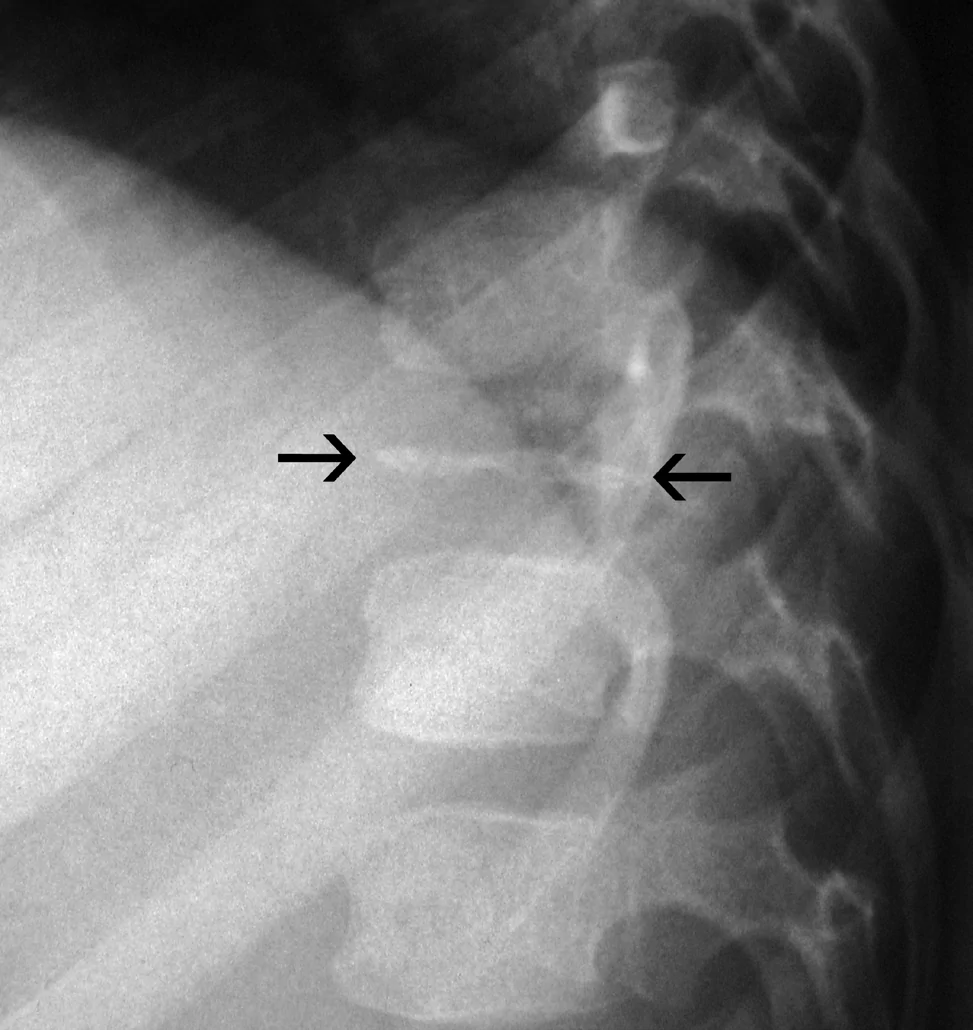
Classic vertebra plana of the lower thoracic spine in a 3-year-old boy with LCH. Conventional radiograph showing complete collapse of the vertebral body (straight arrows) with sclerosis of the residual bone.

**Figure 17:**
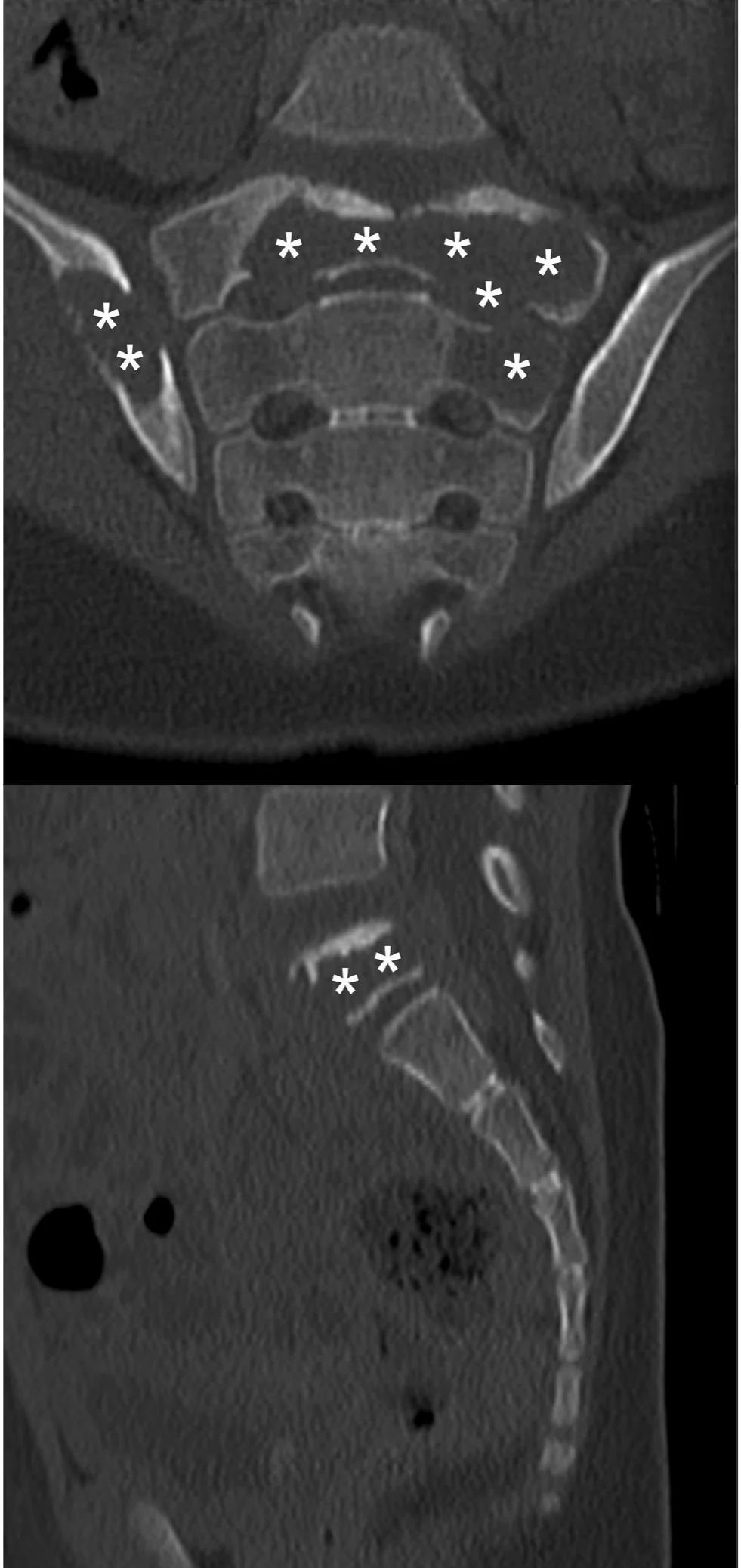
Coronal and sagittal CT images of the sacrum (left and right, respectively) in a 5-year-old girl with LCH. Asterisks demonstrate extensive lytic involvement of S1, S2, and the right ilium, with widespread cortical breach.

**Figure 18:**
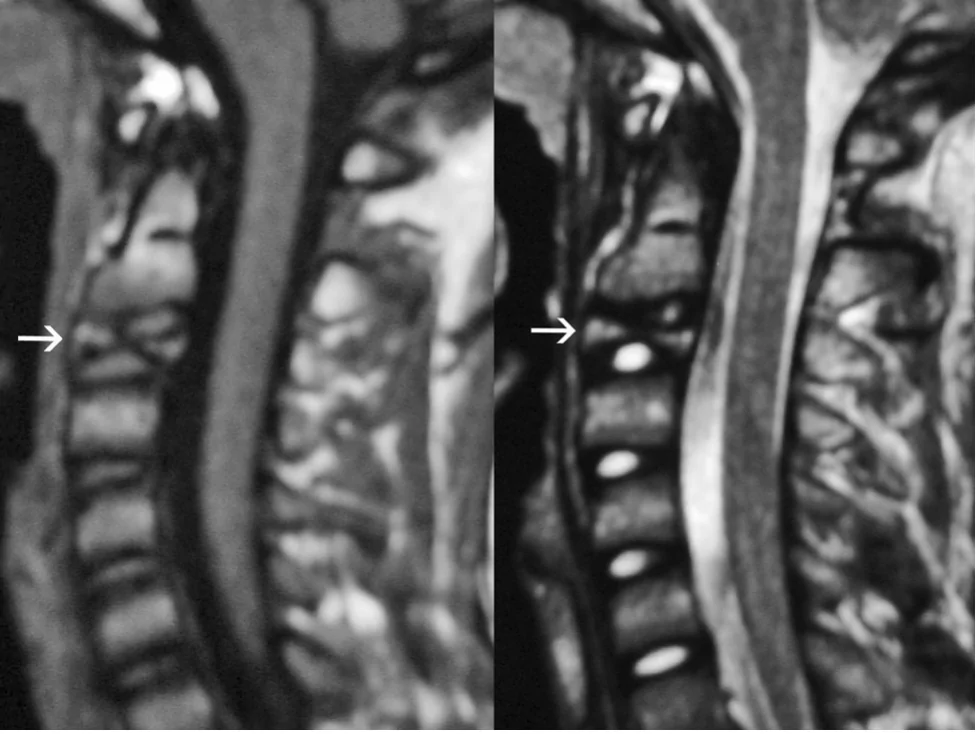
Cervical spine MRI in a 16-year-old boy with treated LCH (T1WI on the left; T2WI on the right), showing partial collapse of C3 (straight arrows), more pronounced centrally, yielding a biconcave configuration, with near-normal signal intensity following treatment. Note the preservation of the adjacent intervertebral discs.

##### CNO/CRMO

At-a-glance—Demographic profile: school age to adolescence; Segment: predominantly thoracic, often multifocal; Portion: vertebral body (endplates/corners) with posterior-element involvement common.

For baseline mapping and follow-up of CNO/CRMO, WBMRI is preferred; classic extra-spinal foci in the metaphyses of the long bones often clinch the diagnosis when interpreted alongside the spinal pattern—particularly when the presentation is spine-predominant or spine-only^**(^16^–^19^)**^. In children, clavicular disease is typically isolated to the clavicle—most often the medial third with lateral progression—while sparing the sternoclavicular joint and sternum^**(^16^–^19^)**^. An MRI of the spine typically shows marrow edema that is hypointense on T1WI and hyperintense on STIR sequences, together with endplate/corner changes and mild paraspinal reaction with preserved discs (Figure 19); abscess is atypical^**(^17^,^18^)**^. Complications include pathologic fractures/vertebra plana; unlike LCH, a collapsed vertebra in CNO/CRMO generally does not re-expand, which guides counseling and follow-up^**(^39^)**^. Management escalates from nonsteroidal anti-inflammatory drugs to disease-modifying antirheumatic drugs/biologics, with bisphosphonates effective in vertebral disease; most lesions partially resolve, although there can be new-level recurrence^**(^18^,^40^)**^.

**Figure 19:**
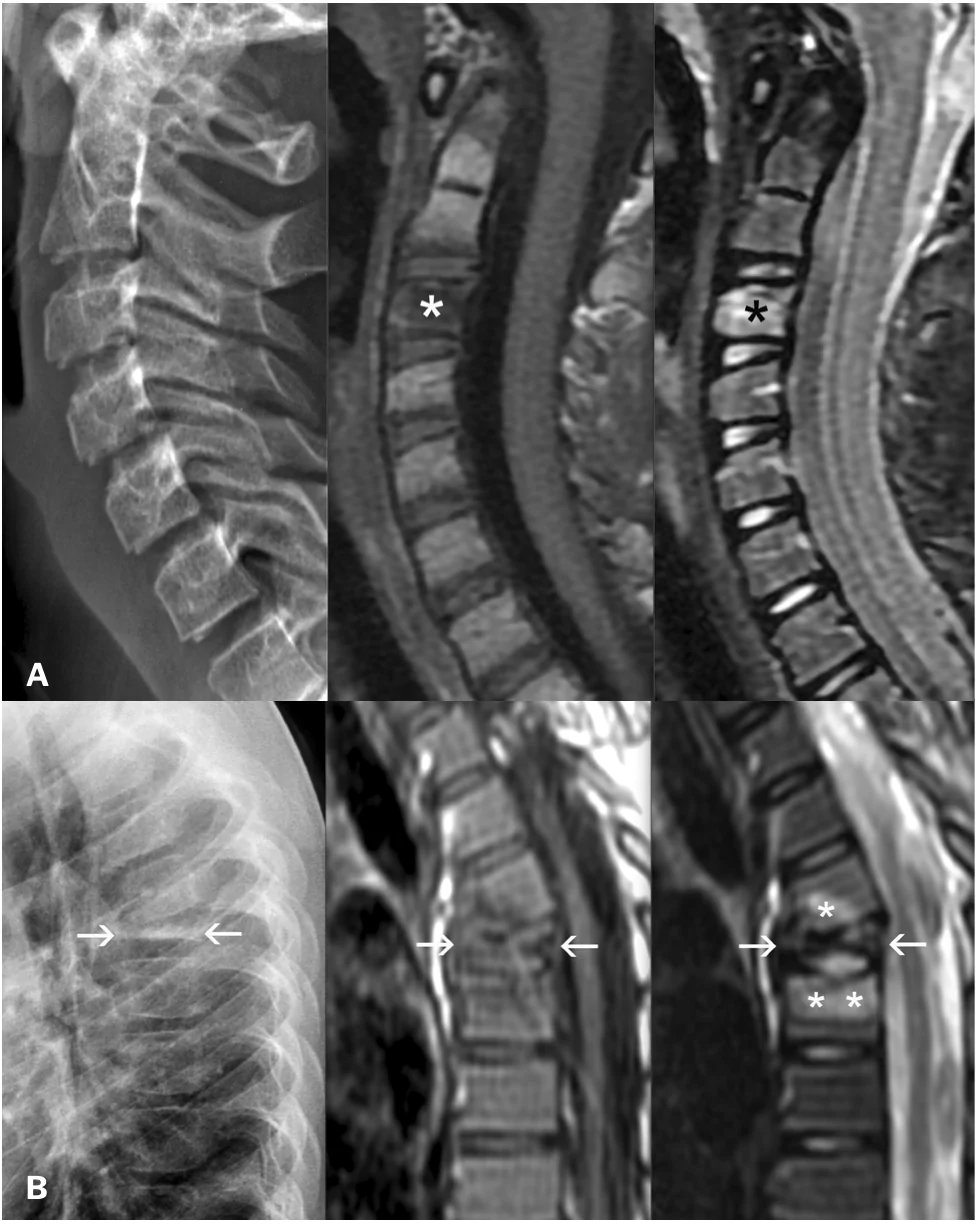
Spectrum of vertebral involvement in CNO. In **A**, lateral radiograph, sagittal T1WI, and sagittal STIR sequence of the cervical spine in a 12-year-old girl, showing isolated, extensive marrow edema in the C3 vertebral body due to osteitis (asterisks), which can mimic diffuse infiltrative processes or post-traumatic change. In **B**, lateral radiograph, sagittal T1-WI, and sagittal STIR sequence of the upper thoracic spine in a 10-year-old boy, showing advanced collapse of the vertebral body (near vertebra plana, straight arrows), with sclerosis on the radiograph and only mild bone marrow edema on MRI, with accentuated thoracic kyphosis. Note the additional marrow edema adjacent to the inferior endplate of the superior vertebra and the superior endplate of the inferior vertebra (asterisks), as well as the preservation of the intervening discs.

##### Fibrous dysplasia

At-a-glance—Demographic profile: children/adolescents (monostotic or polyostotic, consider McCune–Albright features); Segment: variable (spinal involvement is uncommon and usually polyostotic); Portion: body or posterior elements.

Fibrous dysplasia is a fibro-osseous lesion driven by post-zygotic GNAS mutations (encoding the stimulatory G-protein alpha subunit, Gsα), which cause constitutive Gs signaling and replacement of normal bone by fibrous tissue and disorganized woven bone^**(^42^)**^. Conventional radiography and CT show a ground-glass matrix with smooth expansion and a sclerotic rim; MRI findings are variable and nonspecific, with low-to-intermediate signal strength on T1WI and intermediate-to-high signal strength on T2WI (Figure 20), depending on trabeculae and cystic/hemorrhagic change^**(^5^,^10^)**^. In the spine, monostotic disease is rare; most vertebral cases are polyostotic, and management ranges from observation to stabilization for deformity/instability^**(^42^)**^.

**Figure 20:**
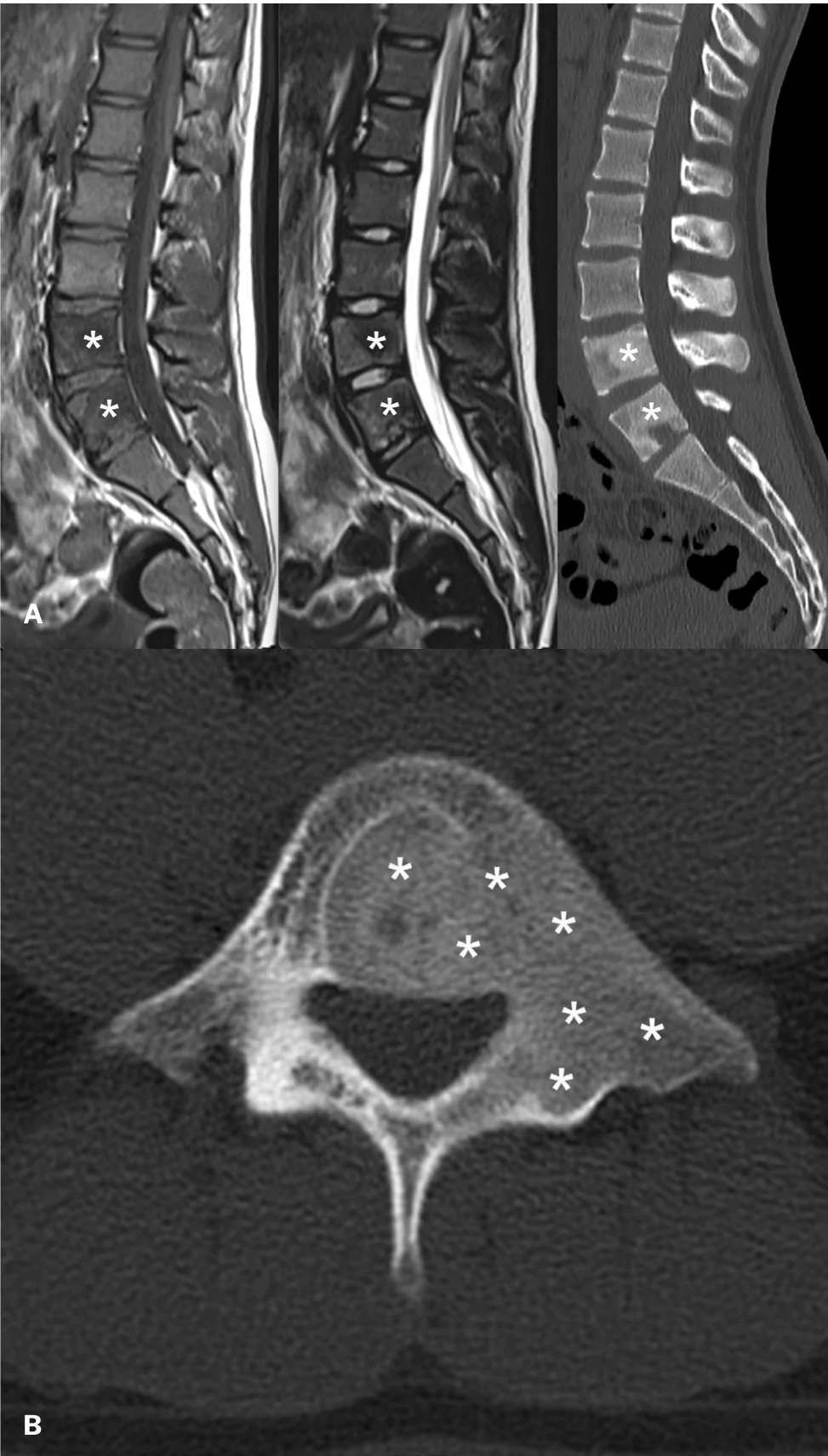
Polyostotic fibrous dysplasia in a 15-year-old boy. In A, sagittal T1WI and T2WI MRI scans (left and center, respectively) showing altered marrow signal intensity within the L4 and L5 vertebral bodies (asterisks), predominantly hypointense relative to the normal upper lumbar vertebrae, without bone destruction, expansion, or soft-tissue mass. Sagittal CT (right, in A) showing well-circumscribed areas of increased bone density corresponding to those regions of low signal intensity (asterisks). The characteristic homogeneous ground-glass matrix is best appreciated on axial CT (asterisks, in B), on which it can be seen to be surrounded by a sclerotic rind. A focal lucency adjacent to the L5 inferior endplate, poorly characterized on MRI, illustrates the variable imaging appearance of fibrous dysplasia.

## CONCLUSION

Primary vertebral tumors and tumor-like lesions in skeletally immature patients are heterogeneous and frequently imaging-driven diagnoses. A structured, modality-wise approach—grounded in pediatric anatomy and growth, precise compartmental localization, and judicious use of radiography, CT, MRI, and WBMRI—enables timely, accurate differentiation among malignant, benign, and tumor-like entities^**(^1^–^4^)**^. Recognizing pediatric-specific signatures—including vertebra plana with potential height reconstitution in LCH versus lack of re-expansion in CNO/CRMO; posterior-element bias and the broad yet condensed spectrum of osteoblastoma; WBMRI patterns in CNO/CRMO—and applying updated terminology and concepts refine the interpretation and management^**(^16^–^19^,^23^,^24^,^36^–^38^)**^.

## Data Availability

Not applicable.
